# ASSOCIATIONS BETWEEN HEALTH AND POSITIVE AFFECT IN SPOUSAL DEMENTIA CARE DYADS

**DOI:** 10.1093/geroni/igac059.1574

**Published:** 2022-12-20

**Authors:** Joan Monin, Kalisha Bonds Johnson

**Affiliations:** Yale School of Public Health, New Haven, Connecticut, United States; Emory University, Atlanta, Georgia, United States

## Abstract

Little is known about the conditions that foster greater positive affect in the daily lives of spousal dementia care dyads in the early stages of dementia. This study aimed to examine the extent to which multiple indicators of health, including activities of daily living needs, quality of life, and the person with dementia’s behavioral symptoms were associated with each partner’s positive affect in daily life. Using secondary baseline data from a randomized controlled trial testing a stress reduction intervention in 63 couples (N=126), we examined whether individuals’ multiple health indicators were associated with their own positive affect (actor effects) and their partner’s positive affect (partner effects). Actor partner interdependence model results showed that for both persons with dementia and spouses, actor quality of life was the greatest predictor of positive affect, controlling for all other actor and partner health indicators (β=.04, SE=.01, t(67.1)=3.36, p=.001).

